# Heme-binding protein 1 delivered via pericyte-derived extracellular vesicles improves neurovascular regeneration in a mouse model of cavernous nerve injury

**DOI:** 10.7150/ijbs.81809

**Published:** 2023-05-11

**Authors:** Jiyeon Ock, Jitao Wu, Fang-Yuan Liu, Fitri Rahma Fridayana, Lashkari Niloofar, Minh Nhat Vo, Soon-Sun Hong, Ju-Hee Kang, Jun-Kyu Suh, Guo Nan Yin, Hai-Rong Jin, Ji-Kan Ryu

**Affiliations:** 1 National Research Center for Sexual Medicine and Department of Urology, Inha University College of Medicine, Incheon, 22332, Republic of Korea.; 2 Department of Urology, Yantai Yuhuangding Hospital Affiliated to Medical College of Qingdao University, Yantai, Shandong 264000, People's Republic of China.; 3 Department of Biomedical Sciences, College of Medicine, Program in Biomedical Science & Engineering, Inha University, Incheon, Korea.; 4 Department of Pharmacology, Medicinal Toxicology Research Center, Inha University College of Medicine, Incheon, Korea.

**Keywords:** Cavernous nerve injury, Peripheral nerve injury, Heme-binding protein 1, Extracellular vesicles, Neurovascular regeneration

## Abstract

As a peripheral nerve injury disease, cavernous nerve injury (CNI) caused by prostate cancer surgery and other pelvic surgery causes organic damage to cavernous blood vessels and nerves, thereby significantly attenuating the response to phosphodiesterase-5 inhibitors. Here, we investigated the role of heme-binding protein 1 (Hebp1) in erectile function using a mouse model of bilateral CNI, which is known to promote angiogenesis and improve erection in diabetic mice. We found a potent neurovascular regenerative effect of Hebp1 in CNI mice, demonstrating that exogenously delivered Hebp1 improved erectile function by promoting the survival of cavernous endothelial-mural cells and neurons. We further found that endogenous Hebp1 delivered by mouse cavernous pericyte (MCP)-derived extracellular vesicles promoted neurovascular regeneration in CNI mice. Moreover, Hebp1 achieved these effects by reducing vascular permeability through regulation of claudin family proteins. Our findings provide new insights into Hebp1 as a neurovascular regeneration factor and demonstrate its potential therapeutic application to various peripheral nerve injuries.

## Introduction

Heme-binding protein 1 (Hebp1) is an intracellular tetrapyrrole-binding protein that is possibly involved in heme or porphyrin biosynthesis.^1^ Recent studies have demonstrated the importance of Hebp1 in the central nervous system, especially in neurodegenerative disease.^2^, ^3^ Moreover, a previous study showed that N-terminal cleavage of Hebp1 by cathepsin D produces a 21-amino acid peptide called F2L that may promote neutrophil migration.^4^ In addition, a genome-wide transcriptome analysis and functional studies conducted by Yin GN et al. suggested that Hebp1 promotes neurovascular regeneration in diabetes-induced erectile dysfunction (ED) by restoring endothelial cell and neural cell contents and reducing reactive oxygen species (ROS) levels.^5^, ^6^ However, the cellular and molecular mechanisms underlying these actions of Hebp1 in the peripheral nervous system are still poorly understood.

Although neurogenic ED accounts for only 10% to 19% of ED cases,^7^, ^8^ peripheral nerve injuries, such as cavernous nerve injury (CNI) during prostate cancer surgery and other pelvic surgeries, results in CNI-induced ED in more than 80% of patients,^9^ severely affecting the quality of life of men and their partners. Although considerable research has been devoted to exploring new methods or targets of neuroprotection and nerve regeneration for saving erectile function in ED patients,^10^-^15^ severe cavernous neurovascular damage limits the efficacy of first-line treatments for CNI-induced ED, such as oral phosphodiesterase-5 (PDE5) inhibitors.^8^ Therefore, additional new therapeutic strategies are needed for regeneration of neurovascular function in CNI-induced ED.

Extracellular vesicles (EVs), which are secreted by cells into the extracellular space and are thought to play key roles in delivering various lipids, proteins and nucleic acids, are involved in intercellular communication and exchange of biological information among cells.^16^-^18^ EVs have been investigated for their therapeutic potential in regenerative medicine applications utilizing animal disease models of cardiovascular, kidney, hepatic, and neurological diseases based on their size, contents and manner of production.^19^, ^20^ Recent studies have shown that EVs play important roles in many processes, such as apoptosis, cell proliferation, differentiation, migration, angiogenesis, oxidative stress, aging, and inflammation.^21^, ^22^ Furthermore, although multiple studies have shown that pericyte-derived EV-mimetic nanovesicles (PC-NVs) may ameliorate ED induced by CNI, diabetes mellitus, or Peyronie's disease in mouse models and promote peripheral nerve regeneration in a mouse model of sciatic nerve injury,^23^-^26^ the biological components of EVs and mechanisms through which they promote tissue repair and regeneration remain unknown.

The present study sought to evaluate the efficacy of Hebp1 in improving ED in CNI mice. First, we found that Hebp1 levels in the penis were reduced in CNI-induced ED mice. We further demonstrated that intracavernous delivery of Hebp1 protein improved erectile function by promoting the survival of cavernous endothelial-mural cells and neurons. Hebp1 protein-enriched mouse cavernous pericyte-derived EVs (MCP-EVs) also improved erectile function by rescuing vascular and neurological abnormalities in CNI-induced ED mice. Notably, these latter effects were abolished by Hebp1 knockdown in MCP-EVs. Finally, Signaling Explorer Antibody Arrays showed that Hebp1 delivered by MCP-EVs significantly regulated claudin family proteins in CNI-induced ED mice.

## Results

### Decreased expression of Hebp1 in the penis of CNI-induced ED mice

**Figure 1:**
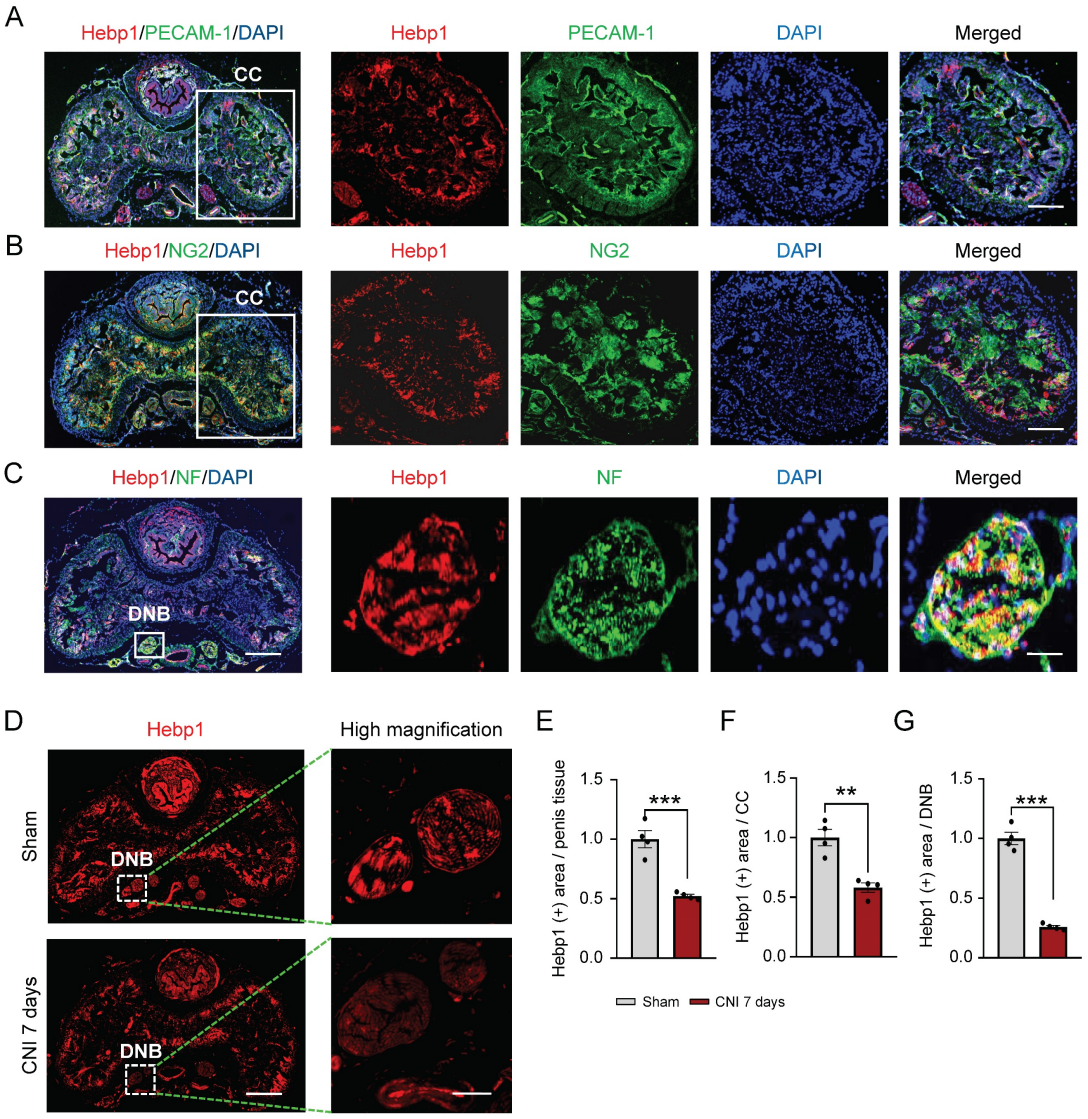
** Hebp1 expression is decreased in the penis of CNI-induced ED mice.** (A-C) Representative (left) and high-magnification (right) images of double-immunofluorescence staining of normal mouse CC and DNB tissue using antibodies against Hebp1 (red), PECAM-1 (A; green), NG2 (B; green), and NF (C; green). Nuclei were labeled with the DNA dye DAPI (blue). Scale bars, 400 µm (left), 100 µm (right top and middle), and 25 µm (right bottom). (D) Representative (left) and high-magnification (right) images of Hebp1 (red) immunofluorescence staining in penis tissues from sham-operated and CNI-induced ED mice (7 days after CNI). (E-G) Quantitative analyses of Hebp1 expression in total penis tissue (E), the CC (F), and DNBs (G) were performed using an image analyzer. Results are presented as means ± SEM (n = 4; **P < 0.01, ***P < 0.001 vs. sham operation groups). The relative ratio of the sham operation group was defined as 1. CC, corpus cavernosum; DNBs, dorsal nerve bundles; ED, erectile dysfunction; CNI, cavernous nerve injury; NF, neurofilament; DAPI, 4,6-diamidino-2-phenylindole.

A previous study showed that Hebp1 protein expression is significantly decreased in diabetic ED mice and that exogenous Hebp1 treatment promotes regeneration of dorsal nerve bundles (DNBs) in this animal model of ED.^5^ Therefore, we hypothesized that Hebp1 is involved in neuronal cell and/or peripheral nerve regeneration. To test this, we first examined the expression of Hebp1 in DNBs from cavernous tissue of sham-operated and CNI-induced ED (7 days) mice. Double-immunofluorescence staining with antibodies against Hebp1 (red) and the endothelial cell marker PECAM-1 (green), pericyte marker NG2 (green), or axonal marker neurofilament-2000 (NF; green) showed that Hebp1 is expressed not only in the corpus cavernosum (CC), but also in DNBs of the normal mouse penis (**Figure 1**A-**1**C). In addition, we found that the expression of Hebp1 was reduced 2-fold in total penis tissue (**Figure 1**D and **1**E) and the CC (**Figure 1**D and **1**F) of CNI-induced ED mice compared with the sham-operated mice, and by up to 4-fold in DNBs of the CNI-induced ED group (**Figure 1**D and **1**G). These results suggest that, in addition to being involved in cavernous angiogenesis, Hebp1 is also likely involved in cavernous nerve regeneration.

### Intracavernous injection of Hebp1 improves erectile function in CNI-induced ED mice

**Figure 2:**
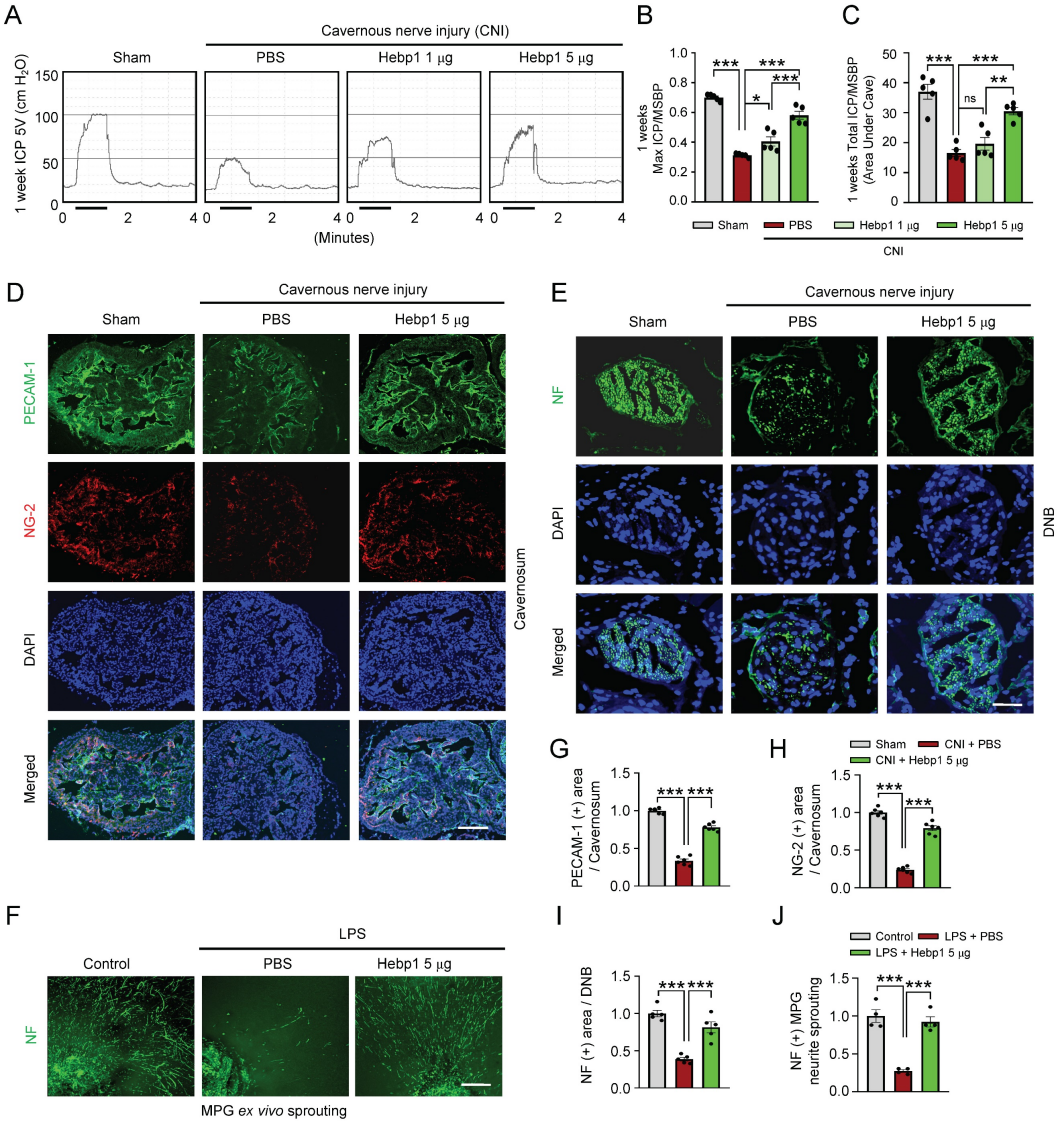
** Hebp1 protein improves erectile function in CNI-induced ED mice.** (A) Representative ICP responses for sham-operated and CNI-induced ED mice stimulated 1 week after two intracavernous injections (administered on days -3 and 0) of Hebp1 protein (1.0 and 5 µg per mouse). The stimulus interval is indicated by a solid bar. (B, C) Ratios of mean maximal ICP and total ICP (area under the curve) to MSBP were calculated for each group. Results are presented as means ± SEM (n = 5). (D, E) Immunofluorescence staining of CC and DNB tissues for PECAM-1 (D; green), NG2 (D; red), and NF (E; green) after ICP studies. Nuclei were labeled with the DNA dye DAPI (blue). Scale bars, 100 µm (D) and 25 µm (E). (F) NF (green) immunofluorescence staining in mouse MPGs exposed to LPS (2.5 μg/ml) and then treated with Hebp1 (5 µg/ml) or PBS for 5 days. Scale bar, 100 μm. (G-I) Quantitative analysis of CC endothelial cell (G, PECAM-1), pericyte (H, NG2) and neuronal cell (I, NF) contents using an image analyzer. Results are presented as means ± SEM (n = 5). (J) Neurite sprouting, quantified using an image analyzer. Results are presented as means ± SEM (n = 4; *P < 0.05, **P < 0.01, ***P < 0.001). The relative ratio of the sham operation or control group was defined as 1. ED, erectile dysfunction; CNI, cavernous nerve injury; NF, neurofilament; DAPI, 4,6-diamidino-2-phenylindole; MPG, major pelvic ganglion; PBS, phosphate-buffered saline; LPS, lipopolysaccharide; MSBP, mean systolic blood pressure; DNB, dorsal nerve bundles; ns, not significant.

To determine whether Hebp1 also has beneficial effects on erectile function in CNI-induced ED mice, we assessed erectile function in sham-operated and CNI-induced ED mice that received two intracavernous injections (administered on days -3 and 0) of different concentrations of Hebp1 protein (1, 5 or 10 µg per mouse in 20 µl PBS) or phosphate-buffered saline (PBS). During electrical stimulation, the ratios of maximal and total intracavernous pressure (ICP) to mean systolic blood pressure (MSBP) were significantly reduced in control (PBS-treated) CNI-induced ED mice compared with sham-operated mice. Notably, repeated intracavernous injections of Hebp1 in CNI-induced ED mice significantly improved this erection parameter, restoring 82% of sham operation group values at 5 µg/mouse and partially improving erectile function at 1 µg/mouse (**Figure 2**A-**2**C). However, at higher concentrations, Hebp1 protein caused a strong inflammatory response in penile tissue; therefore, we were unable to obtain functional results at a concentration of 10 µg/mouse. No significant differences in body weight or MSBP were found among experimental groups. Immunofluorescence staining for PECAM-1, NG2, and NF in CC tissue showed that Hebp1 significantly reestablished endothelial cell contents (**Figure 2**D and **2**G), including phosphorylated endothelial NO synthase (eNOS) (**Figure S1**), as well as contents of pericytes (**Figure 2**D and **2**H) and nerves (**Figure 2**E and **2**I), in CNI-induced ED mice. We next attempted to determine the effect of Hebp1 on neurite outgrowth in major pelvic ganglion (MPG) explants exposed to lipopolysaccharide (LPS). Immunofluorescence staining of MPG explants for NF showed a significant reduction in neurite sprouting in control (PBS treated) MPG explants exposed to LPS; in contrast, Hebp1 treatment significantly enhanced neurite outgrowth in LPS-exposed explants (**Figure 2**F and **2**J). These results indicate that Hebp1 promotes restoration of cavernous endothelial-mural cell and neuronal cell contents, thereby improving erectile function in CNI-induced ED mice.

### Hebp1 restores pericyte contents by reducing apoptosis of pericytes and increasing their proliferation in CNI-induced ED mice

**Figure 3:**
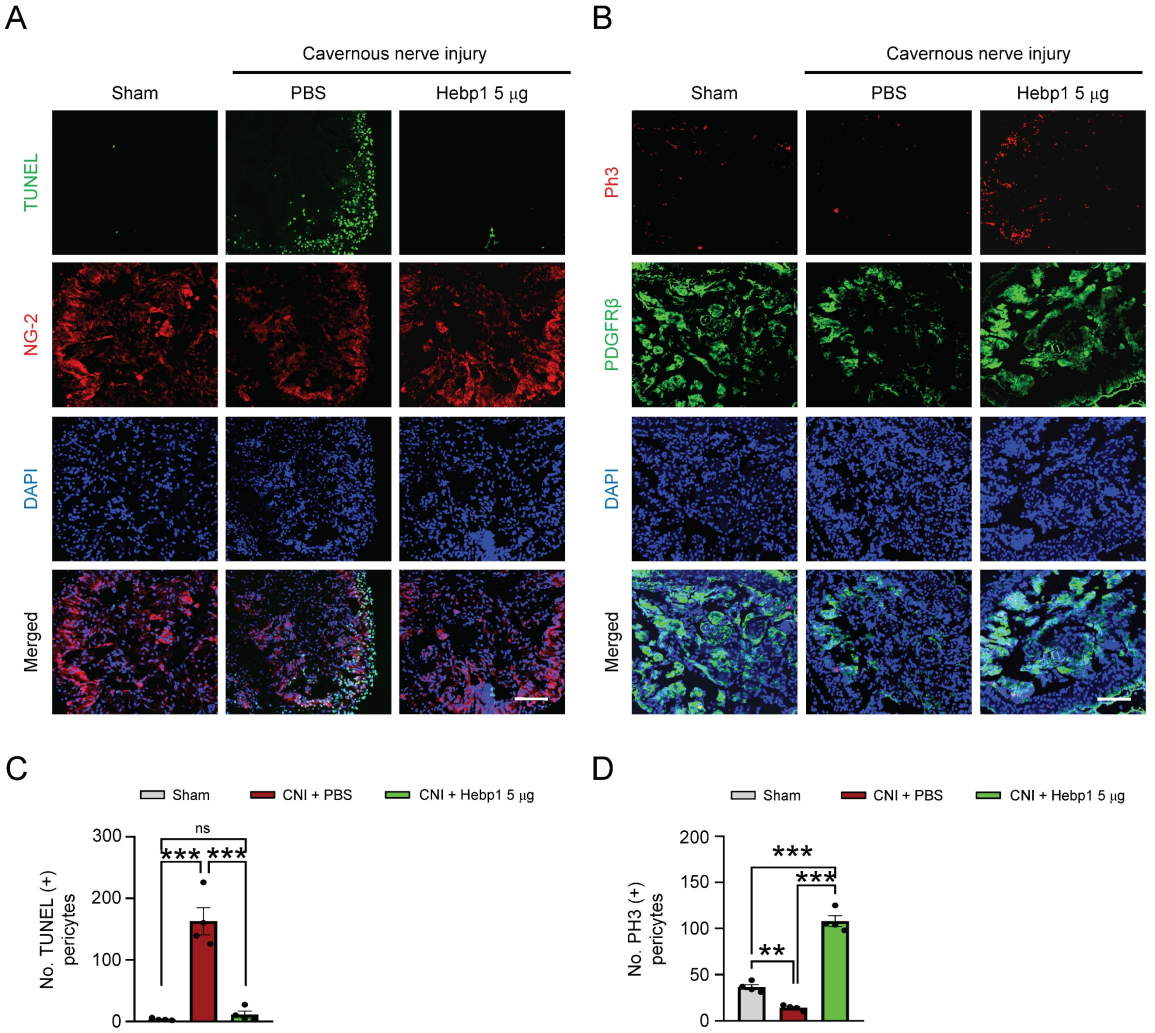
** Hebp1 protein reduces apoptosis of pericytes and induces their proliferation in the penis of CNI-induced ED mice.** (A) TUNEL assay (green) and NG-2 (red) immunofluorescence staining of mouse cavernous tissues in sham-operated and CNI-induced ED mice 1 week after two intracavernous injections (administered on days -3 and 0) of Hebp1 protein (5 µg per mouse in 20 µl). Nuclei were labeled with the DNA dye DAPI. Scale bar, 100 μm. (B) Immunofluorescence staining of CC tissue for phospho-histone H3 (PH3; red) and PDGFRβ (green) using the same samples as above. Scale bar, 100 μm. (C, D) Number of TUNEL-positive (C) or PH3-positive (D) pericytes, quantified using an image analyzer. Results are presented as means ± SEM (n = 4). TUNEL, terminal deoxynucleotidyl transferase-mediated deoxyuridine triphosphate nick end labeling; DAPI, 4,6-diamidino-2-phenylindole; ED, erectile dysfunction; CNI, cavernous nerve injury; PBS, phosphate-buffered saline; ns, not significant.

A previous study showed that Hebp1 expression was higher in mouse cavernous pericytes than in endothelial cells.^5^ To determine whether exogenous Hebp1 could restore the contents of pericytes in CC tissue of CNI-induced ED mice, we assessed apoptosis (TUNEL assay) and proliferation (PH3 staining) of pericytes in CC tissue of CNI-induced ED mice. We found that apoptosis of mouse cavernous pericytes was significantly increased and proliferation was severely reduced in control (PBS-treated) CNI-induced ED mice; however, apoptosis returned to normal levels in CNI-induced ED mice treated with Hebp1 (**Figure 3**A and **3**C), and proliferation levels increased to twice that of the sham operation group (**Figure 3**B and **3**D). Taken together, these data suggest that Hebp1 restores pericyte contents in CNI-induced ED mice by reducing apoptosis and increasing pericyte proliferation.

### Hebp1 delivered by MCP-EVs contributes to nerve regeneration

**Figure 4:**
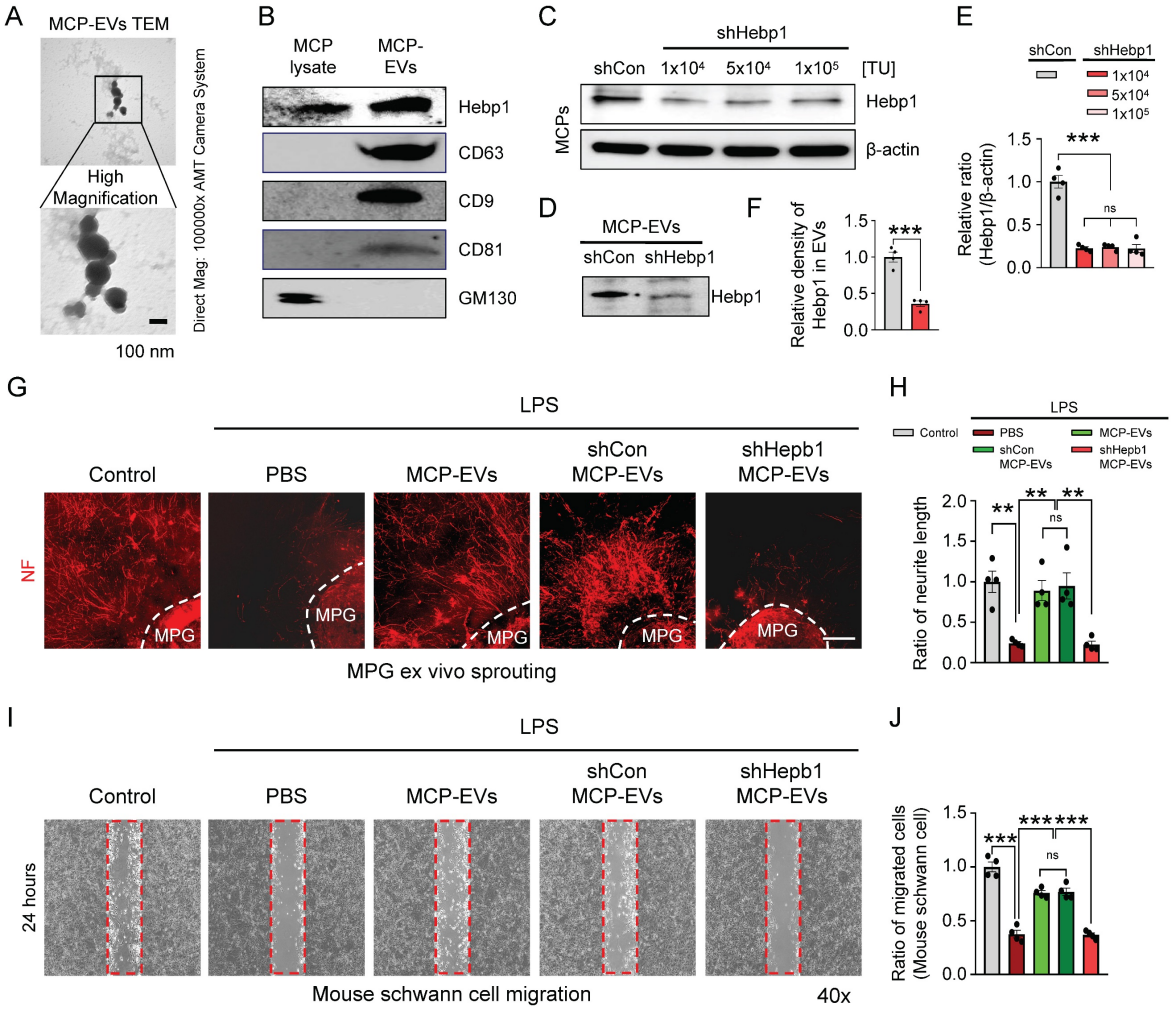
** Hebp1 is delivered by MCP-EVs and contributes to nerve regeneration.** (A) Representative transmission electron microscopy (TEM) phase images for detecting isolated MCP-EVs. Scale bar = 100 nm. (B) Representative Western blot for Hebp1, EV positive markers (CD63, CD9 and CD81) or EV negative marker (GM130) in MCPs and MCP-EVs. (C, D) Expression of Hebp1 in MCP-EVs infected with lentivirus expressing shHebp1 or shCon at three doses (1 × 10^3^, 5 × 10^4^, and 1 × 10^4^ TU/ml culture medium) for at least 3 days. (E, F) Densitometric quantification of Hebp1 protein bands using an image analyzer. Results are presented as means ± SEM (n = 4). (G, H) Immunofluorescence staining for NF in mouse MPG tissues exposed to LPS (2.5 μg/ml) and other indicated conditions for 5 days (G). Lengths of NF-positive neurites in MPG tissues, quantified using an image analyzer (H). Results are presented as means ± SEM (n = 4). Scale bar, 100 µm. (I, J) Migration assays of mouse Schwann cells exposed to LPS (2.5 μg/ml) and other indicated conditions for 24 hours (I). Number of migrated cells in the red dotted rectangle, quantified using an image analyzer (J). Results are presented as means ± SEM (n = 4; **P < 0.01, ***P < 0.001). The relative ratio of the shCon (control) group was defined as 1. MCPs, mouse cavernous pericytes; EVs, extracellular vesicles; LPS, lipopolysaccharide; MPG, major pelvic ganglion; PBS, phosphate-buffered saline; ns, not significant.

It is well known that EVs deliver numerous cargoes that may play important roles in cellular communication, including mRNAs, miRNAs, lipids, and proteins.^16^, ^17^ Although a previous study showed that Hebp1 is derived from MCPs and can induce angiogenesis,^5^ the detailed delivery pathways remain unclear. As a first step to defining this pathway, we isolated and characterized MCP-EVs (**Figure 4**A and **4**B) from the culture medium of MCPs, we found that MCPs-EVs are around 100 nm in size (**Figure 4**A). And through DiD-labeled MCPs-EVs tracking analysis, we found that the concentration of DiD-labeled MCPs-EV showed a decreasing trend in the CC tissues, which was completely absorbed at 24 hours. And the amount of DiD-labeled MCPs-EVs transferred to dorsal nerve bundles (DNBs) was the highest at 12 hours (**Figure S2**). Next, we assessed Hebp1 levels in MCP-EVs and found that they contained a large amount of Hebp1 protein (**Figure 4**B). To evaluate whether Hebp1 delivered by MCP-EVs contributes to nerve regeneration* in vitro*, we inhibited Hebp1 expression in MCPs by infection with lentivirus expressing small interfering RNA (siRNA) against Hebp1 (shHepb1) and then isolated Hebp1-depleted MCP-EVs for MPG neurite sprouting assays and Schwann cell migration assays; scrambled small hairpin RNA (shCon) was used as a control (**Figure 4**B-**4**E). Under LPS-exposed conditions, shCon MCP-EVs, with intact Hebp1, significantly enhanced neurite outgrowth, restoring it to normal levels; however, treatment with Hebp1-depleted EVs derived from shHebp1-treated MCPs (shHepb1 MCP-EVs) had no such effect (**Figure 4**F and **4**G). Schwann cell migration assays further showed that shHepb1 MCP-EVs could not promote Schwann cell migration (**Figure 4**H and **4**I).

**Figure 5:**
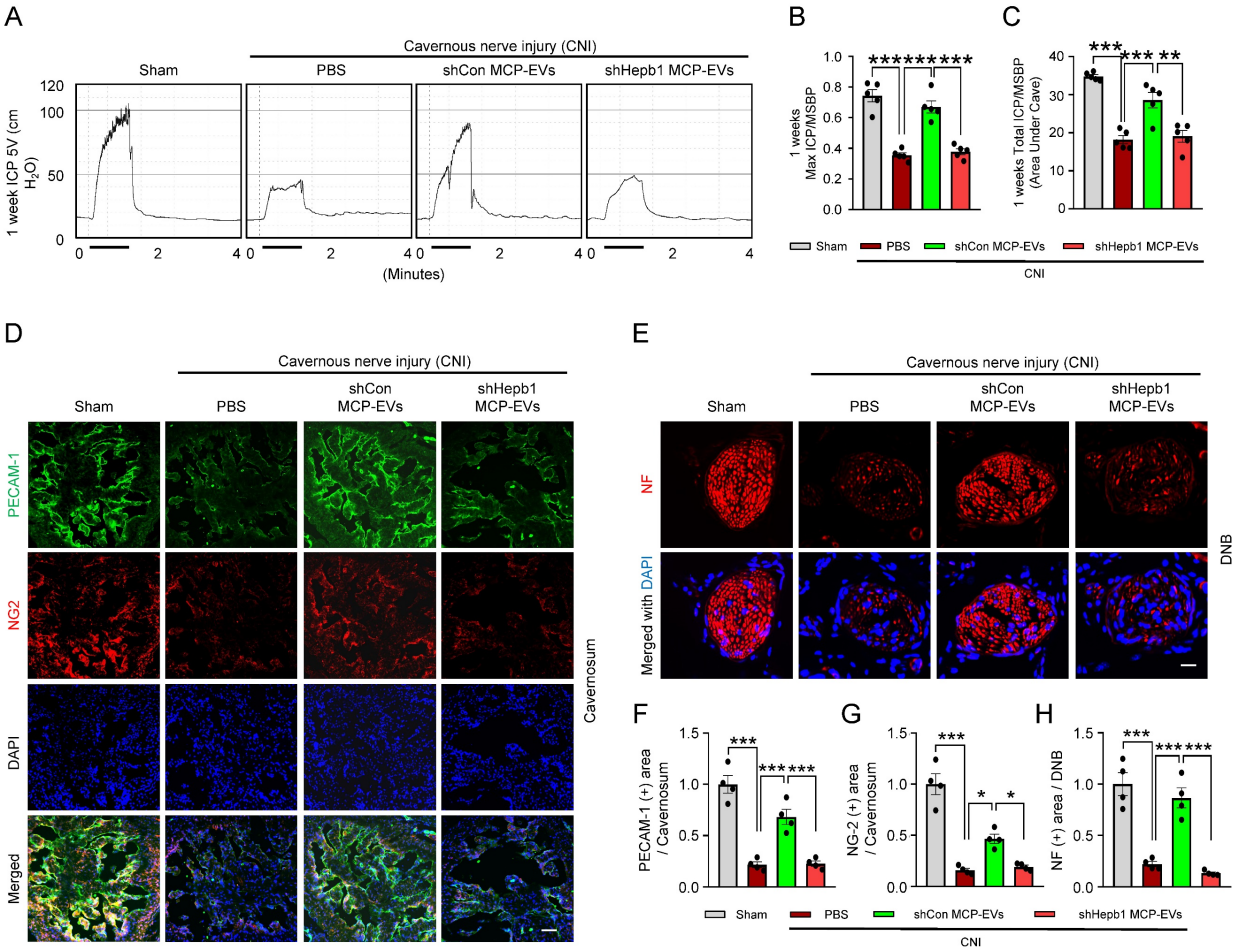
** Hebp1 is delivered by MCP-EVs and contributes to nerve regeneration.** (A) Representative ICP responses for sham-operated or CNI-induced ED mice stimulated 1 week after two intracavernous injections (administered on days -3 and 0) of shCon MCP-EVs (10 µg per mouse in 20 µl PBS), shHebp1 MCP-EVs (10 µg per mouse in 20 µl PBS), or PBS only. The stimulus interval is indicated by a solid bar. (B, C) Ratios of mean maximal ICP and total ICP (area under the curve) to MSBP, calculated for each group. Results are presented as means ± SEM (n = 5). (D, E) Immunofluorescence staining for PECAM-1 (D; green), NG2 (D; red), and NF (E; green) in CC and DNB tissues after ICP studies. Nuclei were labeled with the DNA dye DAPI (blue). Scale bars, 100 µm (D) and 25 µm (E). (F-H) Quantitative analysis of CC endothelial cell (F, PECAM-1), pericyte (G, NG2) and neuronal cell (H, NF) contents using an image analyzer. Results are presented as means ± SEM (n = 4; *P < 0.05, **P < 0.01, ***P < 0.001). The relative ratio of the sham operation group was defined as 1. ED, erectile dysfunction; MCPs, mouse cavernous pericytes; EVs, extracellular vesicles; PBS, phosphate-buffered saline; MSBP, mean systolic blood pressure; DAPI, 4,6-diamidino-2-phenylindole; DNB, dorsal nerve bundles; ns, not significant.

Next, to assess the nerve-regeneration effects of Hebp1 delivered by MCP-EVs in CNI-induced ED mice *in vivo*, we evaluated erectile function in sham-operated and CNI-induced ED mice that received two intracavernous injections of shHebp1 MCP-EVs (10 µg per mouse in 20 µl PBS), shCon MCP-EVs (10 µg per mouse in 20 µl PBS) or PBS only, administered on days -3 and 0. Treatment with shCon MCP-EVs significantly improved the ratio of maximum ICP or total ICP to MSBP in CNI-induced ED mice, yielding values comparable to those in the sham operation group. By contrast, these erectile function improvement effects were absent in CNI-induced ED mice treated with shHebp1 MCP-EVs (**Figure 5**A-**5**C). Consistent with this, immunofluorescence staining for PECAM-1, NG2, and NF in CC tissue revealed that shCon MCP-EVs significantly improved endothelial cell (**Figure 5**D and **5**F), pericyte (**Figure 5**D and **5**G), and nerve (**Figure 5**E and **5**H) contents in CNI-induced ED mice, thereby ameliorating erectile dysfunction. No significant differences in body weight or MSBP were found among experimental groups. Collectively, these results indicate that MCP-EVs containing Hebp1 (i.e., shCon MCP-EVs) are able to promote neurovascular regeneration in CNI-induced ED mice.

### Identification of the signaling pathway mediated by MCP-EV-delivered Hebp1 responsible for neurovascular regeneration in CNI-induced ED mice

**Figure 6:**
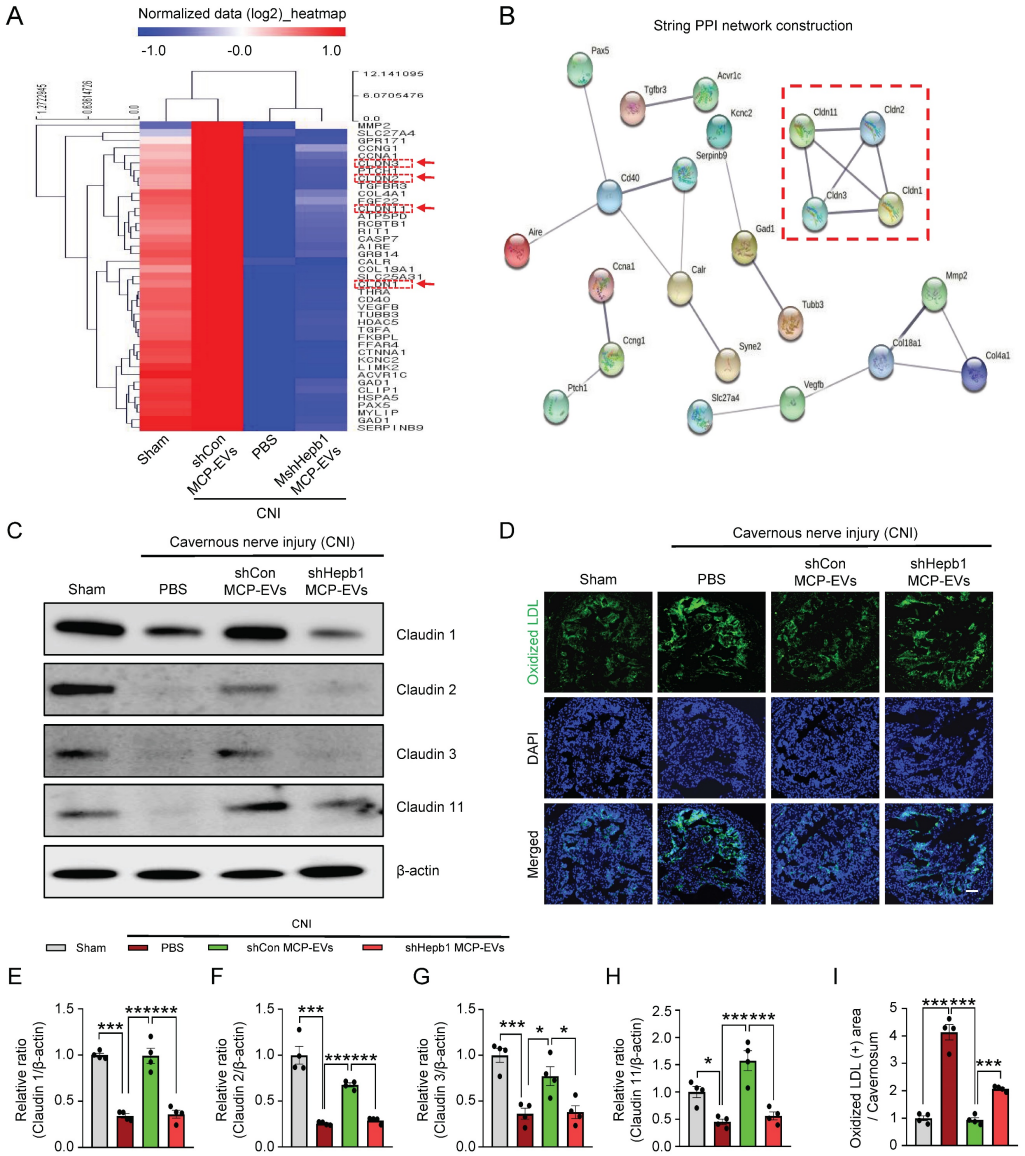
** Identification of the signaling pathway mediating the effects of MCP-EV-delivered Hebp1 in CNI-induced ED mice.** (A) Heatmap of log2-normalized intensities of significantly differentially expressed genes in Signaling Explorer Antibody Arrays from sham-operated and CNI-induced ED mice that received two intracavernous injections (administered on days -3 and 0) of shHebp1 MCP-EVs (10 µg per mouse in 20 µl PBS), shCon MCP-EVs (10 µg per mouse in 20 µl PBS), or PBS only. Color density shows fold-changes, with different multiples indicated by different colors. Red, up-regulated; blue, down-regulated. (B) Proteins significantly regulated by MCP-EV-delivered Hebp1 in CNI-induced ED mice, identified by a PPI network of differentially expressed genes. The thickness of lines corresponds to the strength of the interaction between the proteins. Red box highlights claudin family genes. (C) Representative Western blots for claudin -1, -2, -3, and -11 in CC tissue from the indicated groups. (D) Ox-LDL (green) immunostaining in CC tissues from the indicated groups. Nuclei were labeled with the DNA dye DAPI (blue). Scale bar, 100 µm. (E-I) Expression of the indicated proteins, quantified by densitometric analysis of the corresponding protein bands, and Ox-LDL immunopositive areas, determined using an image analyzer. Results are presented as means ± SEM (n = 4; *P < 0.05, ***P < 0.001). The relative ratio of the sham operation group was defined as 1. ED, erectile dysfunction; MCPs, mouse cavernous pericytes; EVs, extracellular vesicles; PBS, phosphate-buffered saline; Ox-LDL, oxidized low-density lipoprotein; DAPI, 4,6-diamidino-2-phenylindole; ns, not significant.

To further elucidate the signaling pathway that mediates MCP-EV-delivered Hebp1 actions, we performed Signaling Explorer Antibody Array profiling of 1358 unique antibodies covering 20 cell signaling pathways in mouse cavernosum tissues from sham-operated and CNI-induced ED mice injected with shHebp1 MCP-EVs or shCon CP-EVs. Mice in each group received two intracavernous injections of shHebp1 MCP-EVs (10 µg per mouse in 20 µl PBS) , shCon MCP-EVs (10 µg per mouse in 20 µl PBS) or PBS only, administered on days -3 and 0. Among the 1358 protein targets, only 41 (**Table S1**) showed contra-regulation (≥3-fold ratio) between the two groups; specifically, expression levels of these proteins increased in the CNI + shCon MCP-EV group compared with the CNI + PBS group, but were significantly decreased in the CNI + shHebp1 MCP-EV group (**Figure 6**A). Next, to analyze the protein-protein interaction (PPI) network, we used the STRING database, which integrates known and predicted PPIs for predicting protein functional interactions. This PPI network analysis identified the claudin family proteins, claudin-1, -2, -3, and -11 as being significantly regulated by MCP-EV-delivered Hebp1 in CNI-induced ED mice (**Figure 6**B). Western blot analyses further confirmed these results (**Figure 6**C and **6**E-**6**H). However, by immunoprecipitation experiments, we found that only claudin-2 and claudin-3 were confirmed to be regulated by binding to Hebp1 directly, while claudin-1 and claudin-11 were not regulated by binding to Hebp1 (**Figure S3**). In addition, immunofluorescence staining revealed that vascular permeability, measured as extravasation of oxidized-LDL (**Figure 6**D and **6**I), and reactive oxygen species (ROS) production, determined by hydroethidine staining for superoxide anions and nitrotyrosine staining for peroxynitrite detection, (**Figure S4**) were significantly reduced in the CNI + shCon MCP-EV group, effects that were entirely lacking in the CNI + shHebp1 MCP-EV group. Taken together, these findings suggest that vascular permeability and ROS signaling constitute key process involved in mediating the effects of MCP-EV-delivered Hebp1 in CNI-induced ED mice.

## Discussion

**Figure 7:**
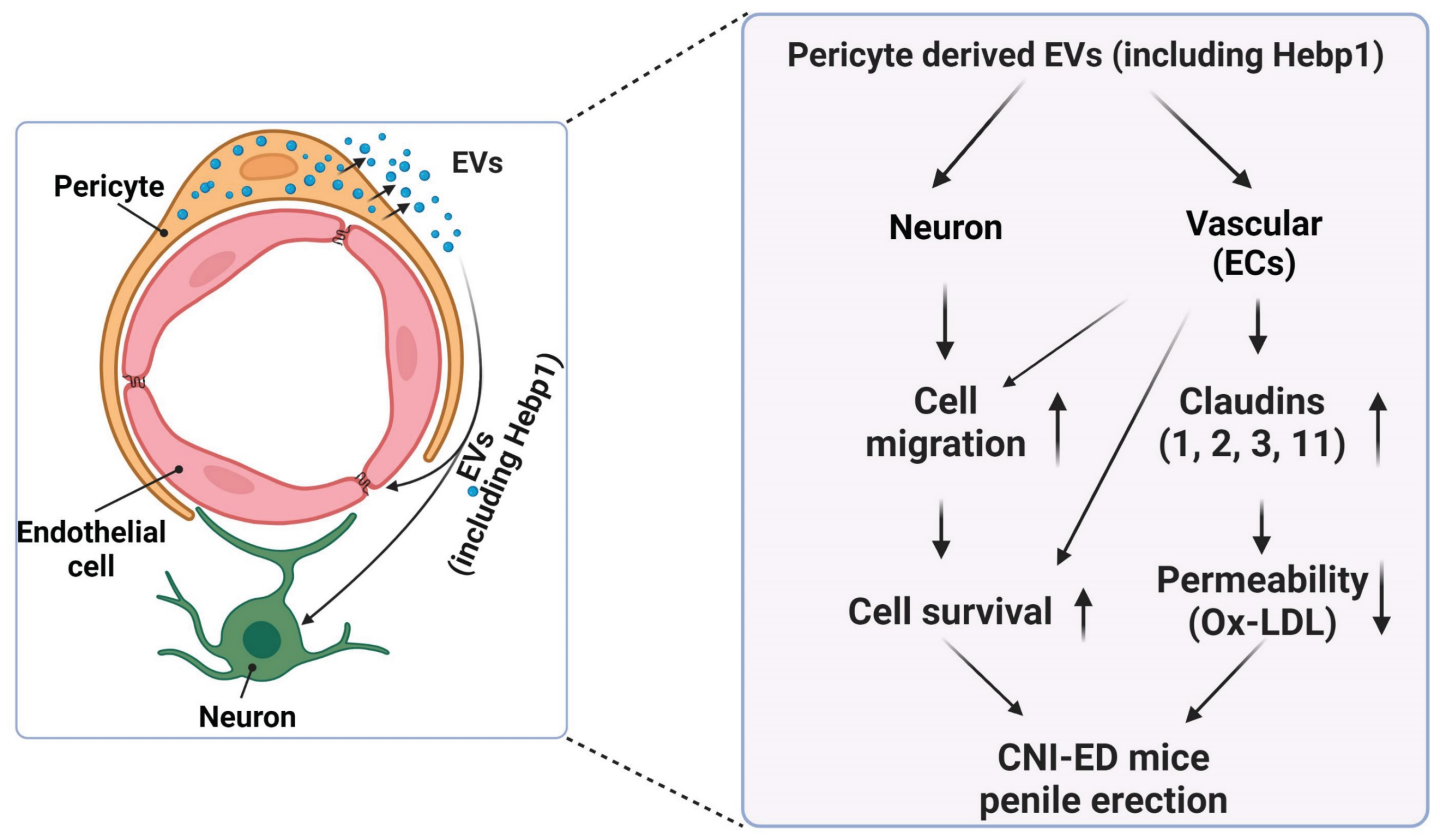
** Schematic depiction of the proposed mechanism for MCP-EV-delivered Hebp1 in CNI-induced ED mice.** EVs, extracellular vesicles; ECs, endothelial cells; Ox-LDL, oxidized low-density lipoprotein; CNI, cavernous nerve injury; ED, erectile dysfunction.

A review of the literature indicates that relatively few articles on Hebp1 have been published in the last 20 years. As an intracellular tetrapyrrole-binding protein, Hebp1 is known to be involved in heme regulation, biosynthesis, and transport.^1^, ^27^ A recent study that screened for proteomic changes associated with early Alzheimer's disease (AD) stages by Yagensky et al. demonstrated that Hebp1 is predominantly expressed in neurons, interacts with the mitochondrial contact site complex, and is involved in heme-induced neuronal death in AD.^3^ However, the biological functions of Hebp1 in the peripheral nervous system remain unclear. Our recent genome-wide transcriptome analyses and animal studies demonstrate that Hebp1 derived from MCPs can rescue cavernous endothelial cell and neuronal cell contents and modulate ROS levels and PI3K/AKT/eNOS signaling activity in a mouse model of diabetes-induced ED.^5^, ^25^ In the absence of reports on Hebp1 in peripheral nerve injury, we hypothesized that Hebp1 plays a beneficial role in CNI-induced ED. Findings presented in the current study demonstrate that Hebp1 delivered by MCP-EVs promotes penile neurovascular regeneration in CNI-induced ED mice and suggest that this action reflects regulation of claudin family proteins. **Figure 7** summarizes the proposed mechanisms of action of Hebp1 delivered by MCP-EVs in ameliorating diabetic ED.

To test our hypothesis, we first assessed the expression of Hebp1 in cavernous tissue of sham-operated and CNI-induced ED mice. We found that Hebp1 expression was significantly decreased in CNI-induced ED mice compared with sham-operated mice, suggesting that the expression of Hebp1 is related to nerve injury; notably, this decrease was greater in DNBs than in the CC. We further found that exogenously delivered Hebp1 protein effectively restored the contents of cavernous endothelial-mural cells and neuronal cells, and ultimately improved erectile function in CNI-induced ED mice, particularly MPG neurite sprouting and MCP survival. ICP/MSBP ratio, another parameter for measuring erectile function, was also improved by Hebp1 treatment at a dose of 5 μg/mouse; however, ICP results could not be obtained at higher doses (10 μg/mouse) owing to severe inflammation. These findings suggest that local injection of Hebp1 may be a promising strategy for treatment of CNI-induced ED, with potential application to the treatment of other peripheral nerve injuries. However, how endogenous Hebp1 is transmitted and functions remains unknown. In addition, we measured the absorption spectra of Hebp1 and hemin (**Figure S5**) to determine whether the Hebp1 we used contained heme and performed MPG neurite sprouting under the indicated conditions to see if the presence of heme affected Hebp1 function (**Figure S6**). We found that high doses of heme (10 μM) reduced neurite sprouting, and even treatment with Hebp1 did not restore neurite sprouting, which may be related to the severe cell death induced by high doses of heme rather than the function of Hebp1 being affected.

A close relationship between pericyte dysfunction and ED, especially ED caused by diabetes and nerve injury, has been recently reported.^28^ For example, DKK2 (dickkopf WNT signaling pathway inhibitor 2) mediates the interaction between pericytes and endothelial cells and promotes neurovascular regeneration in diabetic ED through the angiopoietin (Ang1)-Tie2 pathway.^14^ In addition, PC-NVs promote penile erection in diabetic ED mice in a lipocalin 2 (Lcn2)-dependent manner.^23^ However, studies of the role of pericytes in ED are still in their infancy and details are lacking. A number of new candidate mediators of the effects of pericyte-derived EVs have been provided by genome-wide transcriptome analyses of MCPs.^6^ Among these, Hebp1—the most attractive candidate—was found to exert neurovascular-regeneration effects when exogenously delivered in CNI-induced ED mice. Collectively, we hypothesized that the beneficial effects of endogenous Hebp1 reflect its transport to the site of the nerve injury through MCP-EVs. Because EVs are physically extracted from cells or tissues, they are easier to apply to translational medicine, which are derived from natural secretions in the body. Therefore, we assessed the expression of Hebp1 in MCP-EVs. Consistent with our hypothesis, we found that Hebp1 was highly expressed in MCP-EVs, which could effectively promote neurovascular regeneration in CNI-induced ED mice. Importantly, this effect was significantly reduced in Hebp1-depleted MCP-EVs. These findings suggest that MCP-EVs can act as intercellular delivery vehicles to transfer Hebp1 to recipient cells and tissues, thereby exerting neurovascular regeneration effects.

We further explored the detailed mechanisms by which Hebp1 delivered by MCP-EVs promotes neurovascular regeneration in CNI-induced ED mice by performing Signaling Explorer Antibody Arrays and PPI network integration (known and predicted PPIs) using the STRING database. Previous studies have shown that expression of claudin-1, -5 and -19, as well as occludin, are significantly reduced in sciatic nerve injury.^29^ Interestingly, our antibody arrays revealed that the tight junction proteins, claudin-1, -2, -3, and -11 are all regulated by MCP-EV-delivered Hebp1. However, our immunoprecipitation studies suggest that Hebp1 may associate with claudin-2 and claudin-3 and regulate their expression, but the expression of claudin-1 and claudin-11 may be regulated through other indirect pathways. These need to be further verified in future experiments. Finally, western blot analysis and extravasation of oxidized-LDL support the conclusion that Hebp1 improves neurovascular regeneration by regulating the expression of tight junction proteins, thereby reducing vascular permeability and improving blood-nerve barriers in cavernous tissues of CNI-induced ED mice.

In summary, this study is the first to evaluate the potential role of Hebp1 in promoting neurovascular regeneration in CNI-induced ED model. Notably, we demonstrate that Hebp1 can be delivered to target cells or tissues via MCP-EVs and is capable of improving neurovascular regeneration. Among the limitations of this study were our inability to demonstrate why Hebp1 functions differently in the central nervous system (early stage of AD) and peripheral nervous system (CNI-induced ED). It is possible that this difference reflects structural changes in Hebp1 during transport via MCP-EVs or cleavage by some other unknown factors, although verifying this will require further research. Our study provides new insights into the function of Hebp1, not only in the context of peripheral nerve disease, but also in the setting of other neurovascular disorders.

## Materials and Methods

### Ethics statement and study design

All experiments were approved by the Institutional Animal Care and Use committee of Inha University (Assurance Number: 200309-691). Male C57BL/6 mice (8 weeks old, 20-25 g; Orient Bio, Inc., Seongnam, Korea) were used in this study. Animal were monitored daily for health and behavior as previously described.^15^, ^32^ In all experiments, animals were anesthetized with an intramuscular injection of ketamine (100 mg/kg; Yuhan Corp., Seoul, Korea) and xylazine (5 mg/kg; Bayer Korea, Seoul, Korea). Animals were euthanized by 100% CO_2_ gas replacement; cessation of heartbeat and respiration were confirmed prior to harvesting tissue. To investigate the efficacy and mechanism of Hebp1 in restoring erectile function in CNI-induced ED mice, we injected recombinant mouse Hebp1 protein (Ca# MBS1372966, Mybiosource, San Diego, California, USA) into the penis of CNI-induced ED mice. Detailed mechanisms were assessed using Signaling Explorer Antibody Arrays (Cat# SET100, Fullmoon Biosystems, Sunnyvale, CA, USA) on penile tissue from CNI-induced ED mice; MPG tissue and Schwann cells (Ca# M1700-57, Sciencell Research Laboratories, San Diego, California, USA) were cultured in the presence of 2.5 μg/ml LPS (Sigma-Aldrich, St. Louis, MO, USA). No mice died during any experiments, and all experiments were performed in a blinded manner.

### CNI-induced ED mouse model and treatment

Mice were anesthetized with ketamine (100 mg/kg) and xylazine (5 mg/kg). The CNI-induced ED mouse model was prepared by crushing the cavernous nerves using a non-serrated hemostat (Karl Stortz Co., Tuttlingen, Germany) as described previously.^30^ Specifically, a hemostat was applied to both side of the cavernous nerve 1 mm distal to the ganglion for 2 minutes with tips fully closed. The sham operation (control group) consisted of exposing the cavernous nerves without directly manipulating them. All treatments were performed immediately after CNI by intracavernous injection. A vascular clamp (Ca# JD-S-101, JEUNG DO BIO& PLANT CO.LTD, Seoul, Korea) was used to compress the base of the penis before injection and was left in place for 30 minutes to prevent back outflow from the penis. Functional and other studies were performed 1 week after CNI.

### Cell culture and treatment

Primary culture of MCPs followed a previously described protocol.^28^, ^31^ Briefly, cavernous tissue was cut into several 1-mm pieces, which were allowed to settle by gravity into collagen I-coated 35-mm cell culture dishes (Ca# 356456, BD Biosciences, San Jose, CA, USA). After incubation of samples with 300 µl complete Dulbecco's modified Eagle Medium (DMEM; Ca# 11995-073, Gibco, Carlsbad, CA, USA) supplemented with 10% fetal bovine serum (FBS, Ca# 16000-044, Gibco), 1% penicillin/streptomycin (Ca# 15070-063, Gibco), and 10 nM human pigment epithelium-derived factor (PEDF; Ca# SRP4988, Sigma-Aldrich) for 15 minutes at 37°C, 900 µl complete medium was added and samples were further incubated at 37°C in a 5% CO_2_ atmosphere, with media changes every 2 days. After cells reached confluence (~2 weeks after the start of culture), sprouting cells only were sub-cultured and then seeded onto dishes coated with 50 µl/ml collagen I (Ca# 5056, Advanced BioMatrix, Carlsbad, CA, USA) for subsequent experiments. Cells were used for experiments between passages 2 and 3.

Mouse Schwann cells were purchased from Sciencell and cultured according to the Sciencell instructions in Schwann cell medium kit (Ca# M1701, Sciencell Research Laboratories) consisting of basal medium, 5% FBS, 1% penicillin/streptomycin and 1% Schwann cell growth supplement at 37°C in a humidified 5% CO_2_ atmosphere. Cells from passages 2 to 5 were used for experiments.

### MCP-derived EV isolation and characterization

MCPs were cultured for 3 days in a T75 flask with 10 ml of medium containing 20% EV-depleted FBS (Ca# a2720801, Gibco). The medium was then collected and EVs from MCPs (MCP-EVs) were isolated using EXOCET exosome isolation solution (Ca# equltra-20a-1, System Biosciences, Palo Alto, CA, USA) according to the manufacturer's instructions. MCP-EVs were resuspended in PBS, filtered through a 0.45-μm filter, and stored at -80°C until further use. MCP-EVs were quantified using an EXOCET exosome quantitation assay kit (Ca# EXOCET96A-1, System Biosciences), and their concentration was adjusted to 1 µg/µl for subsequent experiments.

The morphology and size of the MCP-EVs was detected by transmission electron microscopy (TEM, Electron Microscopy Sciences, Fort Washington, PA, USA) as described previously.^26^ MCP-EVs were characterized by first separating extracted MCP-EVs and whole MCP lysates by SDS-PAGE and probing with antibodies against the positive EV markers CD63 (Ca# 556019, 1:500; Novus Biologicals, Littleton, CO, *USA*), CD9 (Ca# NBP1-00748, 1:500; Novus Biologicals) and CD81 (Ca# NBP1-77039, 1:500; Novus Biologicals), and the negative EV marker GM130 (Ca# 610822, 1:500; BD Biosciences).

MCP-EVs were labeled with red-fluorescent dye 1,1'-dioctadecyl-3,3,3',3'-tetramethylindodicarbocyanine, 4-chlorobenzenesulfonate salt (DiD) (Ca# D7757, Thermo Scientific, Fremont, CA, USA) according to the manufacturer's instructions and as described previously.^23^ 2.5 µL of DiD dye solution was added to 100 µg of MCP-EVs with total 500 µL of PBS. Incubated for 10 min at room temperature, diluted with 9.5 mL of cold PBS, and subjected to ultracentrifugation at 100,000 × g for 2 hours at 4°C. The pellets washed with PBS and re-centrifuged at 100,000 × g for 2 hours. DiD-labeled MCP-EVs pellets then resuspended in PBS and used for tracking analysis. Penis tissues were harvested at indicated time point after injection and fixed with 4% formaldehyde for overnight at 4°C. Tissues were mounted with mounting medium containing the nuclear dye, 4',6-diamidino-2-phenylindole (DAPI; Ca# H1500, Vector Laboratories Inc., Burlingame, CA, USA). DiD dye tracking detection was performed using confocal fluorescence microscopy (K1-Fluo; Nanoscope Systems, Inc., Daejeon, Korea).

For isolation of shRNA-knockdown and control MCP-EVs, MCPs were first infected with lentivirus expressing shRNA targeting mouse Hebp1 (Ca# TL513407V, four unique target-specific 29-mer shRNAs; Origene Technologies, Inc., Rockville, MD, USA) or scrambled control siRNA (shCon) (Origene Technologies, Inc) as described previously.^5^ Media were replaced with fresh media after 2 days, and infected MCPs were maintained for an additional 3 days. MCP-EVs were then isolated using EXOCET exosome isolation solution as described above. The shRNA sequences targeting mouse Hebp1 were as follows: TL513407VC, 5'-CCG TAA CGA GGT CTG GCT TGT GAA GGC AT-3'; TL513407VD, 5'-ATG CCA AGG AAG CAG ACT ATG TTG CTC AT-3'; TL513407VA, 5'-TGT CTC CTA TGA GGA AAG AGC CTG TGA AG-3'; and TL513407VB, 5'-GGT GGC ACC AAT GAC AAA GGA GTC GGC AT-3'. The lentivirus infection was performed in a combined manner (mixed these four unique Hebp1 shRNA). Experiments were performed 3 days after lentivirus infection.

### Measurement of erectile function

Erectile function was measured as described previously.^15^, ^32^ Briefly, mice from each group were anesthetized by intramuscular injection of ketamine (100 mg/kg) and xylazine (5 mg/kg). A bipolar platinum wire electrode (BIOPAC Systems Inc., Goleta, CA, USA) was placed around the cavernous nerve, just distal to the nerve pinching point. Stimulation parameters were as follows: voltage, 5 V; frequency, 12 Hz; pulse width, 1 ms; and duration, 1 minute. Maximal ICP was recorded during tumescence and total ICP was determined from the area under the curve from stimulus onset to 20 seconds after stimulus termination. Systemic blood pressure was measured using a noninvasive tail-cuff system (Visitech Systems, Apex, NC, USA). To normalize changes in systemic blood pressure, we calculated values as the ratio of maximal ICP or total ICP to MSBP.

### Assessment of cell apoptosis in mouse cavernous tissue by TUNEL assay

Apoptosis of pericytes in mouse cavernous tissue was assessed using a TUNEL (terminal deoxynucleotidyl transferase-mediated dUTP nick-end labeling) assay kit (Ca# S7111, Chemicon, Temecula, CA, USA) according to the manufacturer's instructions. After staining, tissue section samples were mounted in a solution containing the nuclear dye, 4,6-diamidino-2-phenylindole (DAPI; Vector Laboratories). Digital images were obtained using a confocal fluorescence microscope (K1-Fluo; Nanoscope Systems, Inc), and the number of TUNEL-positive cells was counted.

### *Ex vivo* neurite sprouting assay

MPG tissue was harvested and maintained as described previously.^15^ Briefly, MPG tissue was isolated from male mice under a dissecting microscope and transferred into sterile vials containing Hank's Balanced Salt Solution (HBSS; Gibco). MPG tissue was then cut into small pieces and plated on 8-well Nunc Lab-Tek Chamber Slides coated with 0.1 mg/ml poly-D-lysine hydrobromide (Sigma-Aldrich). Tissue was covered with Matrigel (Ca# 354234, Becton Dickinson, Mountain View, CA, USA) and then plates were incubated for 5-10 minutes at 37°C in a humidified 5% CO_2_ atmosphere, after which 200 µl of complete Neurobasal medium (Ca# 21103-049, Gibco) supplemented with 2% serum-free B-27 (Ca# 17504-044, Gibco) and 0.5 nM GlutaMAX-I (Ca# 35050-061, Gibco) was added. To mimic the* in vivo* neuroinflammatory conditions of CNI-induced ED, we treated MPG tissue with 2.5 μg/ml LPS (Sigma-Aldrich) immediately after adding medium, as described previously.^30^ The medium was changed every 2 days, and 5 days later, tissue was fixed in 4% paraformaldehyde for at least 30 minutes and neurite outgrowths were immunostained with an anti-neurofilament antibody (Ca# N5389, 1:50; Sigma-Aldrich).

### Cell migration assay

The SPLScar Block system (Ca# 201903, SPL Life Sciences, Pocheon-si, Gyeonggi-do, Korea) was used to generate uniform scratches on 60-mm culture dishes. Pretreated mouse Schwann cells were seeded into the 3-well block at >95% confluence. The block was removed 5 hours later, and the cells were further cultured for 24 hours in Schwann cell medium under the indicated conditions. Images were acquired with a phase-contrast microscope (CKX41; Olympus, Tokyo, Japan), and migrated cells were analyzed by measuring the ratio of cells that moved into the frame line.

### Signaling Explorer Antibody Arrays

Mouse cavernosum tissues from sham-operated and CNI-induced ED mice intracavernously injected (bilaterally) with shHebp1 MCP-EVs, shCon MCP-EVs, or PBS only was used for Signaling Explorer Antibody Assays (Fullmoon Biosystems). This assay, which consists of 1358 unique antibodies covering 20 cell signaling pathways, was performed by E-Biogen (Ebiogen Inc., Seoul, Republic of Korea) as a custom service.

### Clustering and PPI network construction

Hierarchical clustering was performed using the ExDEGA Graphic Plus program (Ebiogen, Inc., Korea) as described previously.^23^ Briefly, clusters and heat maps were visualized with the ExDEGA Graphic plus program, performed as a custom service, using the database STRING (Search Tool for the Retrieval of Interacting Genes/Proteins), which provides a comprehensive protein interactome that includes known and predicted PPI, scored according to their confidence. Interactions in STRING are performed using confidence scores. All networks were visualized and analyzed using the functional protein association networks module in STRING (medium confidence: 0.400) (https://string-db.org).

### Immunoprecipitation

For immunoprecipitation, 500 μg of lysate from mouse penis tissues was incubated with 1-2 μg of the Hebp1 antibody (Ca# NBP2-14977, Novus Biologicals) or normal rabbit IgG antibody (Ca# 2729S, Cell Signaling, Beverly, MA, USA) for 2 h at 4 °C followed by overnight incubation with Protein A/G PLUS-Agarose (Ca# 16-266, Millipore, Temecula, CA, USA) as described previously.^15^ Immunoprecipitates were washed five times with RIPA buffer (Ca# 89900, Thermo Scientific) and then resolved by SDS-PAGE and immunoblotted with the indicated antibodies. Densitometric analyses of western blot bands were performed using an image analyzer system (Image J 1.34; National Institutes of Health [NIH], http://rsbweb.nih.gov/ij/).

### Histological examinations

Mouse CC tissues and MPG explants for immunofluorescence were fixed in 4% paraformaldehyde for 24 hours at 4°C. Frozen tissue sections (15 µm) or *ex vivo* MPG samples were incubated with primary antibodies against the following proteins: Hebp1 (Ca# NBP2-14977, 1:100; NOVUS Biologicals), Hebp1 (Ca# sc-398750, 1:100; Santa Cruz Biotechnology Inc., Dallas, TX, USA), platelet/endothelial adhesion molecule 1 (PECAM-1, Ca# MAB1398Z, 1:100; Millipore), neuron-glial antigen 2 (NG2, Ca# AB5320, 1:100; Millipore), neurofilament (Ca# N5389, 1:50; Sigma-Aldrich), PDGFRβ (Ca# sc-1627, 1:50; Santa Cruz Biotechnology Inc.), phospho-histone 3 (Ca# 06-570, 1:50; Millipore), phospho-eNOS (Ca# PA5-17917, 1:50; Invitrogen, Carlsbad, CA, USA), oxidized low-density lipoprotein (oxidized-LDL, Ca# ab 14519, 1:100; Abcam, Cambridge, UK), or nitrotyrosine (Ca# 06-284, 1:100; Millipore) at 4°C overnight. After several washes with PBS, the samples were incubated with species-appropriate tetramethyl rhodamine isothiocyanate (TRITC)- or fluorescein isothiocyanate (FITC)-conjugated secondary antibodies for 2 hours at room temperature as follows: FITC Affinipure Goat anti-armenian hamster IgG (H+L) (Cat# 127-095-160; 1:100; Jackson ImmunoResearch Laboratories, Inc. West Grove, PA, USA), rhodamine (TRITC) Affinipure Rabbit anti-mouse IgG (H+L) (Cat# 315-025-003; 1:100; Jackson ImmunoResearch Laboratories, Inc.), Donkey anti-Rabbit IgG H&L (DyLight^®^ 550) (Cat# ab98489; 1:100; Abcam), Goat Anti-Rabbit IgG H&L (Alexa Fluor® 488) (Cat# 711-096-152; 1:100; Jackson ImmunoResearch Laboratories, Inc.), Alexa Fluor® 488 AffiniPure Donkey Anti-Mouse IgG (H+L) (Cat# 715-545-150; 1:100; Jackson ImmunoResearch Laboratories, Inc.). Samples were mounted in a solution containing DAPI (Vector Laboratories, Inc.) for nuclei staining. Fluorescence signals were visualized using a K1-Fluo confocal microscope (Nanoscope Systems, Inc). Quantitative histological analyses were performed using an image analyzer system (Image J 1.34; National Institutes of Health [NIH], http://rsbweb.nih.gov/ij/). We normalized the final measured integrated density values using the area values obtained from ImageJ for each image. For each immunofluorescence staining experiment, we evaluate at least four different samples or regions of interest.

### *In situ* detection of superoxide anions

Superoxide anions levels were assessed *in situ* using the oxidative fluorescent dye, hydroethidine (Cat# D11347, 1:5000; Thermo Fisher Scientific), as described previously.^5^ Ethidium bromide fluorescence was quantified using an image analyzer system (Image J 1.34; NIH).

### Western blot analysis

Penis tissue, MCPs, and MCP-EVs were lysed in RIPA buffer (Sigma-Aldrich) supplemented with protease inhibitor cocktail Solution (Ca# P3100-010, GenDEPOT, Katy, TX, USA) and phosphatase inhibitors (Ca# P3200-005, GenDEPOT). An equal amount of protein (50 µg/lane) from these samples were separated by sodium dodecyl sulfate-polyacrylamide gel electrophoresis (SDS-PAGE) on 8-15% gels and then transferred to polyvinylidene difluoride membranes. After blocking with 5% nonfat dry milk for 1 hour at room temperature, membranes were probed with antibodies against Hebp1 (Ca# NBP2-14977, 1:100; NOVUS Biologicals), claudin-1 (Ca# ab242370, 1:1000; Abcam), claudin-2 (Ca# ab53032, 1:1000; Abcam), claudin-3 (Ca# ab15102, 1:1000; Abcam), claudin-11 (Ca# 36-4500, 1:1000; Thermo Fisher Scientific), and/or β-actin (Ca# sc-47778, 1:5000; Santa Cruz Biotechnology Inc.) for 2 hours. After washing three times, signals were visualized using an ECL detection system (Ca# EBP-1073, Bionote Inc., Hwaseong-si, Gyeonggi-do, Korea). Results were quantified densitometrically using an image analysis system (Image J 1.34; NIH).

### Statistical analysis

Results are expressed as means ± SEM of values from at least four independent experiments. Unpaired t-tests were used for comparison of two groups, and a one-way analysis of variance (ANOVA) followed by Tukey's post hoc test was used for comparisons of more than three groups. Analyses were performed using GraphPad Prism version 8 (Graph Pad Software, Inc.), and statistical significance was accepted for P-values less than 0.05.

## Supplementary Material

Supplementary figures and table.
