# Supplementary material for: Heme-binding protein 1 delivered via pericyte-derived extracellular vesicles improves neurovascular regeneration in a mouse model of cavernous nerve injury
Source: Int J Biol Sci. 2023 May 11;19(9):2663–77. doi: 10.7150/ijbs.81809 (PMC10266087; doi:10.7150/ijbs.81809)
Supplement: Supplementary file 1 — Supplementary figures and table. [file ijbsv19p2663s1.pdf]

Supplemental Information

Heme-binding protein 1 delivered via pericyte-derived extracellular vesicles improves neurovascular regeneration in a mouse model of cavernous nerve injury

Jiyeon Ock, Jitao Wu, Fang-Yuan Liu, Fitri Rahma Fridayana, Lashkari Niloofar, Minh Nhat Vo, Soon-Sun Hong, Ju-Hee Kang, Jun-Kyu Suh, Guo Nan Yin, Hai-Rong Jin, and Ji-Kan Ryu

Figure S1

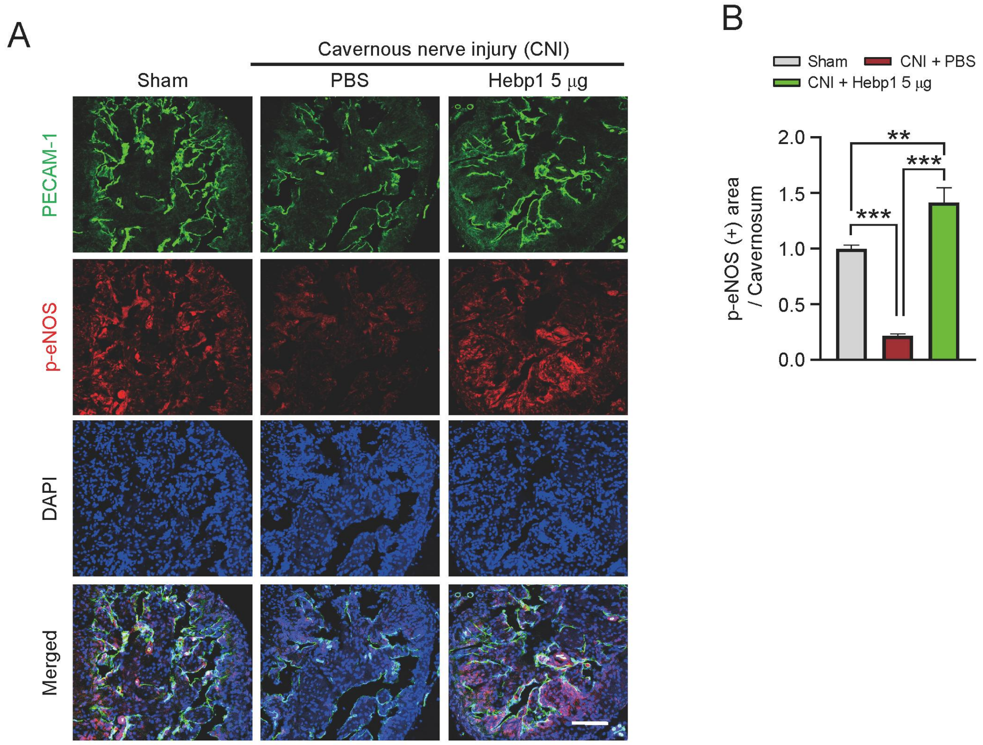

Figure S1. Hebp1 induces cavernous eNOS phosphorylation (p-eNOS) in CNI-induced ED mice.

(A) Double-immunostaining for PECAM-1 (green) and p-eNOS (red) in cavernous tissue from sham operation group or CNI-induced ED mice stimulated at 1 week after two intracavernous injections (administered on days –3 and 0) of Hebp1 protein (5 μg/20 μL). Scale bars, 100 μm. Nuclei were labeled with DAPI (blue). (B) Quantification of p-eNOS–immunopositive area in the cavernosum using an image analyzer. The results are presented as means ± SEM (n = 6). The relative ratio of the sham operation group was defined as 1. ***P < 0.001. DAPI, 4,6-diamidino-2-phenylindole; PBS, phosphate-buffered saline.

Figure S2

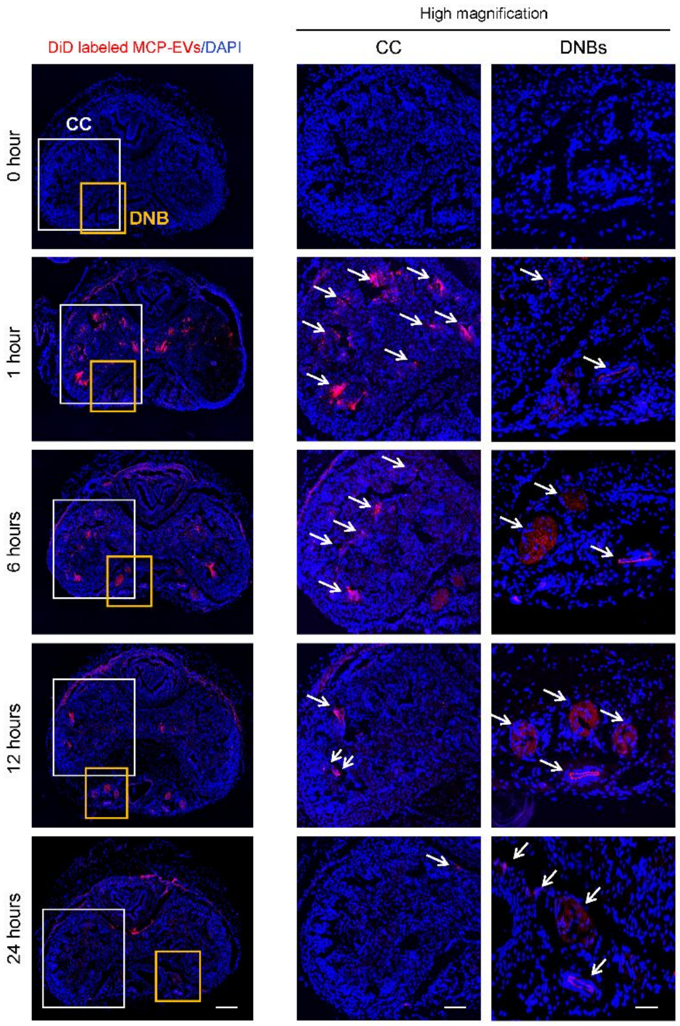

Figure S2: **In vivo detection of DiD-red fluorescently labeled MCPs-EVs in the penis of normal mice.**

The penis tissue was harvested 0, 1, 6, 12, and 24 hours after intracavernous injection of DiD-red labeled MCPs-EVs into the normal mice. DiD-red labeled MCPs-EVs as indicated by the arrows. High magnification images of CC (white frame) and DNBs (orange frame). Nuclei were labeled with the DNA dye DAPI. Scale bars, left, 200 μm; middle, 100 μm; right, 50 μm. CC, corpus cavernosum; DNBs, dorsal nerve bundles; MCP, mouse cavernous pericytes; EVs, extracellular vesicles; DAPI, 4,6-diamidino-2-phenylindole.

Figure S3

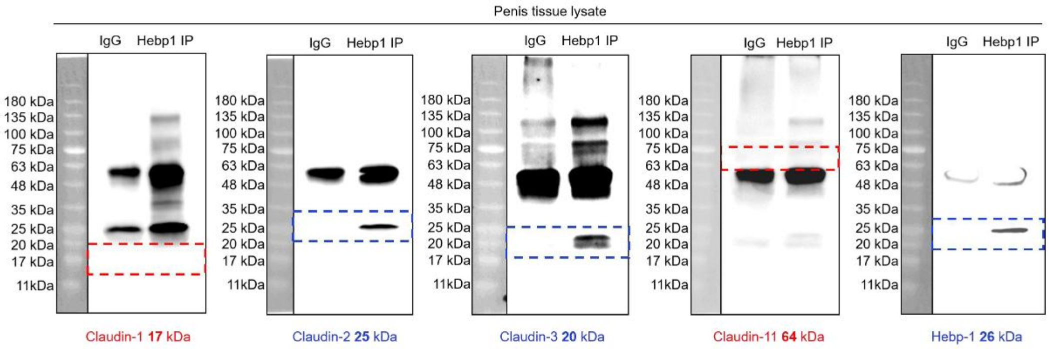

Figure S3. Immunodetection of Hebp1 associated proteins in normal penis tissue lysate.

Immunoprecipitation (IP) of Hebp1 were performed with rabbit anti Hebp1 and rabbit IgG antibodies. Rabbit IgG as negative control. Immunoblot detection of Claudin-1, Claudin-2, Claudin-3, Claudin-11, and Hebp1 were performed. The dotted box indicates the position of the target protein.

Figure S4

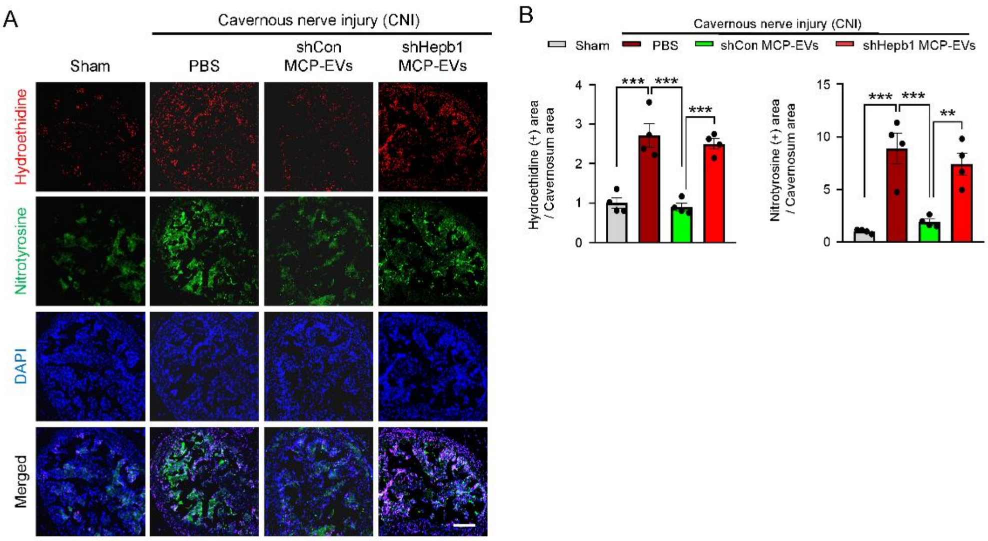

Figure S4. MCP-EVs delivered Hebp1 decreases cavernous ROS production in CNI-induced ED mice.

(A). Double-immunostaining for in situ detection of superoxide anion (hydroethidine, red) and nitrotyrosine production (green) in cavernous tissue from the sham operation group or CNI-induced ED mice stimulated at 1 week after two intracavernous injections (administered on days –3 and 0) of phosphate-buffered saline (PBS), shCon MCP-EVs (10 µg in 20 µl PBS) or shHepb1 MCP-EVs (10 µg in 20 µl PBS). Scale bars, 100 µm. Nuclei were labeled with DAPI (blue). (B). The ethidium bromide fluorescence-immunopositive cavernosum area (B, left) and nitrotyrosine-immunopositive cavernosum area (B, right) were quantified using an image analyzer. The results are presented as means ± SEM (n = 4). The relative ratio of the sham operation group was defined as 1. $^{**}P < 0.01$; $^{***}P < 0.001$. MCP, mouse cavernous pericytes; EVs, extracellular vesicles; DAPI, 4,6-diamidino-2-phenylindole; PBS, phosphate-buffered saline.

Figure S5

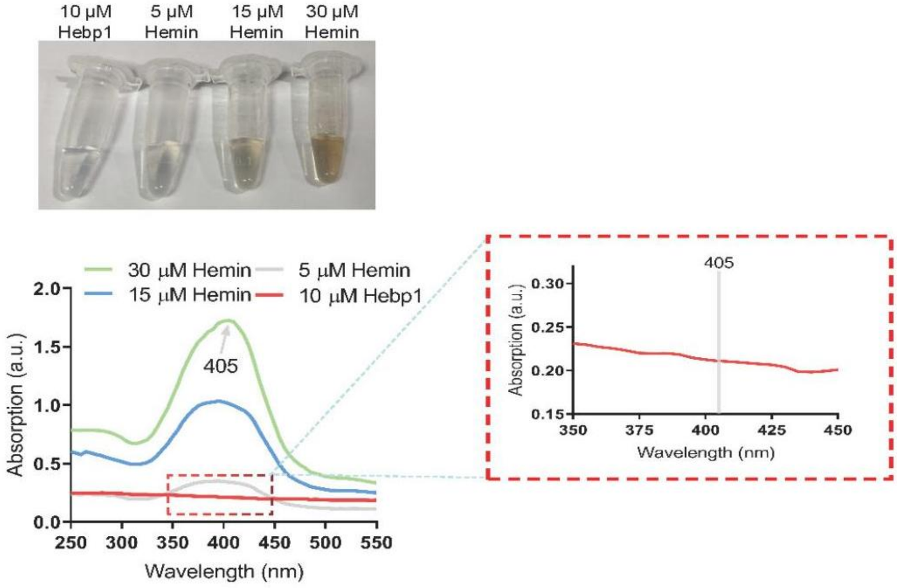

Figure S5. UV-VisibleAbsorption spectra of Hebp1 and Hemin.

Hebp1 (10 µM; ~ 200µg protein) and different concentration of aqueous hemin solution (5,15, and 30 µM; Ca# 51289, Sigma-Aldrich, St. Louis, MO, USA) absorption spectra were measured. The λ_{max} at 405nm was high magnificent as showed right dot frame.

Figure S6

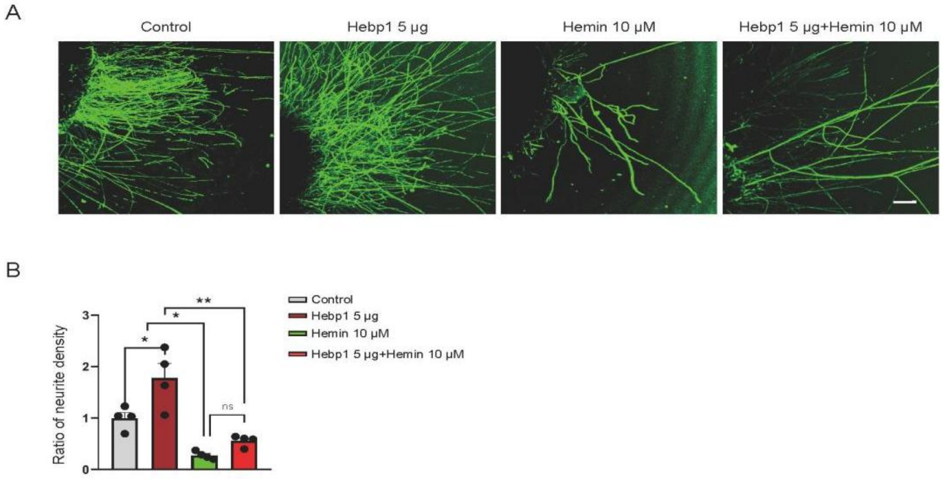

Figure S6. Immunofluorescence staining for mouse MPG tissues.

(A) MPG tissues were exposed to Hebp1 (5 μg/ml), Hemin (10 μM; Ca# 51289, Sigma-Aldrich, St. Louis, MO, USA) and combination conditions. Five days later the MPG tissues were staining with neurofilament (green). (B) Density of neurofilament-positive neurites in MPG tissues, quantified using an image analyzer. Results are presented as means ± SEM (n = 4). Scale bar, 100 μm.

Table S1. Summary of selected contra-regulated targets at least 3 ratios in [CNI+MCP-EVs (shCon)] /CNI or CNI+MCP-EVs (shCon) /CNI+MCP-EVs (shHebp1)

		**Fold change (ratio)**			**Protein information**
**Gene Symbol**	**Reactivity**	**CNI /Sham**	**[CNI+MCP-EVs (shCon)] /CNI**	**CNI+MCP-EVs (shCon) /CNI+MCP-EVs (shHebp1)**	**SwissProt**
**CLDN1**	**H,M,R**	**0.161**	**16.992**	**13.491**	**O95832**
KCNC2	H,M,R	0.195	8.622	7.573	Q96PR1
GPR171	H,M	0.430	7.969	7.354	O14626
**CLDN3**	**H,M,R**	**0.242**	**11.549**	**7.261**	**O15551**
LIMK2	H,M,R	0.181	7.482	6.826	P53671
RCBTB1	H,M	0.198	11.465	6.203	Q8NDN9
FFAR4	H,M,R	0.233	6.265	6.030	Q5NUL3
VEGFB	H,M,R	0.230	7.244	5.872	P49765
PAX5	H,M	0.187	7.577	5.750	Q02548
CLIP1	H,M	0.145	9.541	5.508	P30622
COL18A1	H,M	0.377	5.467	5.283	P39060
SLC27A4	H,M	0.780	4.558	5.214	Q6P1M0
FKBPL	H,M,R	0.252	6.580	5.025	Q9UIM3
PTCH1	H,M	0.321	6.582	5.003	Q13635
TGFA	H,M,R	0.272	6.092	4.716	P01135
MYLIP	H,M	0.221	5.933	4.668	Q8WY64
CCNA1	H,M,R	0.352	6.924	4.423	P78396
**CLDN11**	**H,M,R**	**0.211**	**8.302**	**4.342**	**O75508**
**CLDN2**	**H,M**	**0.390**	**5.364**	**4.303**	**P57739**
SERPINB9	H,M,R	0.172	6.647	4.268	P50453
CASP7	H,M	0.268	6.514	4.174	P55210
TGFBR3	H,M,R	0.401	4.999	4.105	Q03167
CCNG1	H,M,R	0.281	8.406	4.045	P51959
GAD1	H,M,R	0.232	5.800	4.036	Q99259

MMP2	H,M,R	0.678	10.070	3.931	P08253
CTNNA1	H,M,R	0.327	4.197	3.881	P35221
CD40	H,M	0.333	4.548	3.808	P25942
CALR	H,M	0.483	2.994	3.632	P27797
HDAC5	H,M,R	0.327	4.686	3.607	Q9UQL6
TUBB3	H,M,R	0.364	4.315	3.505	Q13509
ATP5PD	H,M,R	0.291	5.597	3.481	O75947
AIRE	H,M	0.317	4.670	3.387	O43918
COL4A1	H,M	0.228	6.311	3.370	P02462
RIT1	H,M	0.364	4.797	3.352	Q92963
GAD1	H,M	0.264	4.207	3.265	Q99259
FGF22	H,M,R	0.230	6.419	3.133	Q9HCT0
HSPA5	H,M,R	0.309	3.898	3.111	P11021
ACVR1C	H,M,R	0.319	3.279	3.093	Q8NER5
GRB14	H,M,R	0.304	4.505	3.087	Q14449
SLC25A31	H,M	0.443	3.285	3.026	Q9H0C2
THRA	H,M,R	0.404	3.481	3.022	P10827
